# The use of Virtual Reality (VR) to assess the impact of geographical environments on walking and cycling: a systematic literature review

**DOI:** 10.1186/s12942-024-00375-6

**Published:** 2024-06-08

**Authors:** Marzieh Ghanbari, Martin Dijst, Roderick McCall, Camille Perchoux

**Affiliations:** 1 Luxembourg Institute of Socio-Economic Research (LISER), Esch-sur-Alzette, Luxembourg; 2 University of Luxembourg, Esch-sur-Alzette, Luxembourg; 3 Luxembourg Institute of Science and Technology (LIST), Esch-sur-Alzette, Luxembourg

**Keywords:** Virtual reality, Walking, Cycling, Experiment, Geographical environments

## Abstract

**Background:**

Geographical environments influence people's active mobility behaviors, contributing to their physical and mental health. The use of Virtual Reality (VR) in experimental research can unveil new insights into the relationship between exposure to geographic environments and active mobility behaviors. This systematic review aims to (1) identify environmental attributes investigated in relation with walking and cycling, using VR, (2) assess their impacts on active mobility behaviors and attitudes, and (3) identify research gaps, strengths and limitations in VR-based experimental research.

**Methods:**

Articles published between January 2010 and February 2022 within five databases (PubMed, Scopus, EBSCO, IEEE Xplore, and Cochrane Library) were explored using three keywords and their synonyms: Virtual Reality, Active mobility behavior, and Geographical environments. Studies focusing on indoor environments, driving simulation, disease-specific groups, non-relevant disciplines (e.g. military, emergency evacuation), VR methodology/software optimization, and those with static participants' involvement were excluded. The full protocol is available from PROSPERO (ID = CRD42022308366).

**Results:**

Out of 3255 articles, 18 peer-reviewed papers met the selection criteria, mostly focusing on walking (83%). Most studies used head-mounted displays (94%) and relied on convenience sampling (72% below 100 participants). Both static (33%) and dynamic (45%) environmental attributes have been investigated, with only 22% of them simultaneously in the same virtual environment. Greenness and crowd density were the most frequent attributes, rather consistently associated with emotional states and movement behaviors. Few studies have taken into account participant’s previous VR experience (33%) and cybersickness (39%) while both are likely to affect an individual’s perception and behavior.

**Conclusions:**

Future research should explore a broader range of environmental attributes, including static and dynamic ones, as well as a more complex integration of these attributes within a single experiment to mimic the effect of realistic environments on people's active mobility behaviors and attitudes. Larger and more diverse population samples are deemed required to improve result generalizability. Despite methodological challenges, VR emerges as a promising tool to disentangle the effect of complex environments on active mobility behaviors.

**Supplementary Information:**

The online version contains supplementary material available at 10.1186/s12942-024-00375-6.

## Introduction

Geographical environments, encompassing built, natural, and social environments, affect people’s active mobility behaviors [1–3]. To design effective interventions that promote active mobility, understanding the intricate relationships between exposure to these environments and engaging in walking and cycling for recreational and mobility purposes is crucial. Experimental research within controlled environments is critical in this attempt, allowing the manipulation of specific attributes and the establishment of causal inference that observational study designs most often fail to document. Virtual Reality (VR) appears as a key methodological tool, enabling researchers to systematically manipulate and examine various attributes of geographical environments while maintaining a high level of realism [4, 5].

VR is a commonly known technology that provides a nearly complete sensory immersion (‘embodied experience’) in a controlled environment [3, 4]. It further enables to create scenarios that would be extremely expensive, risky, or difficult to manipulate in real-life experiments [6, 7] while eliminating the possibility of field-related exogenous confounders [8]. VR has attracted numerous users from many (sub)disciplines including emergency management/evacuation studies [9, 10], wayfinding behavior [11], healthcare and psychology disorders (i.e., stress, anxiety) [8, 12–15], education and training [16], and geography/environmental sciences [17–20]. Its usage notably enables the creation of realistic early-stage experiences for simulating and testing interventions in urban planning. Such early-stage experience is often challenging to grasp with methods such as presenting still images [4, 17, 21, 22]. Additionally, VR is cost-effective, time-efficient, and facilitates easier changes in existing environments compared to post-occupancy surveys, which is a post-experience design evaluation tool [17].

Studies that investigated geographical environments utilizing VR can be divided into three categories: (1) Passive VR experience (i.e., watching an environment) [18], (2) Active VR experience while sitting or standing using a hand controller, joysticks, or buttons to move within a Virtual Environment (VE) [7, 20, 23], and (3) Active VR experience using VR locomotion devices (walking/cycling simulators) [4, 21, 24] or lab-spaces [25–27] to actually walk/cycle in the VE. This review focuses on the last category, as it offers a more immersive experience, sense of embodiment, and aligns with our objective of focusing on active mobility behavior in geographical environments.

Active mobility (i.e., walking and cycling) for recreational and transportation purposes is increasingly recognized for its contributions to physical [28, 29] and mental [30] health, as well as its indirect health benefits such as diminishing car traffic speeds and air and noise pollution [31]. This recognition has led researchers to examine attributes within geographical environments that encourage active mobility behaviors [4]. Furthermore, cities worldwide are recognizing the benefits of active mobility, especially in light of the lessons from COVID-19. They are expanding active mobility infrastructures, and reallocating more public urban spaces to pedestrians and cyclists [32, 33]. Examples of these shifts in urban transportation trends involve bike-sharing programs [34], as well as adopting concepts like Paris’s “15-Minute City” [35].

In exploring active-friendly urban environments, the 5D’s [36] including (1) Density (e.g., building density [31, 37], car density[23], pedestrians’ density [23]), (2) Diversity (e.g., mixed land-use [1, 37–39], (3) Design (e.g., street network characteristics [1, 21, 24, 39, 40], green spaces [1, 6]), (4) Distance: (e.g., distance to public transport [41]), and (5) Destination accessibility (e.g., access networks [37]), and proximity to green spaces [39, 42]), have been stressed as important correlates of active mobility behaviors. However, most of this evidence is based on observational studies. In light of the growing interest in utilizing VR to study environments, and active mobility behavior relationships, it is imperative to conduct a comprehensive systematic review of the research in this area.

This systematic review aims to provide a comprehensive overview of the geographical environment attributes investigated in relation to walking and cycling using VR technology, as well as assess their impact on active mobility behaviors and attitudes. By examining the existing literature, this review pinpoints research gaps, highlights areas for improvement, and outlines both the potential and the ongoing challenges of using VR technology in experimental research in this domain. The findings offer guidance for future research to promote healthier geographical environments.

## Methods

The present systematic review followed the Preferred Reporting Items for Systematic Reviews and Meta-analyses (PRISMA) statement [43]. A protocol was developed and is available from PROSPERO (ID = CRD42022308366).

### Search strategy

A systematic literature search was carried out using five electronic databases, including PubMed, Scopus, EBSCO, IEEE Xplore, and Cochrane Library. The search strategy used a combination of three key elements (a) Virtual reality, its synonyms (e.g., immersive environment) and hardware types used in studies (Head Mounted Display (HMD) and CAVE (Computer Aided Virtual Environment)); (b) Active mobility behavior and its synonyms (e.g., walking and cycling), and (c) Geographical environment and its synonyms (e.g., outdoor environment or urban space). Terms referring to these three keywords were explored in title or abstract words in all databases. A complete list of search terms is available in Appendix 1: Table 1.

### Eligibility criteria

The PICOS framework (Population, Intervention, Comparison, Outcomes, and Study design) was used to formulate eligibility criteria in the systematic review (Table 1). All English-language scientific peer-reviewed papers in the form of original research published between January 2010 and February 2022 were included, while non-peer-reviewed/gray literature (e.g. reports, working papers, book chapters) was excluded.

**Table 1:** PICOS framework with inclusion and exclusion criteria

PICOS	Inclusion criteria	Exclusion criteria
Population	Adults defined as any population aged ≥ 18 years No restriction for gender	Population aged < 18 years Disease-specific groups
Intervention	Exposure to Immersive Virtual Environment (IVE)	Studies focused on non-relevant disciplines Studies in which participants had no physical movement
Comparison	Real vs VR where applicable	Comparing VR types (i.e., HMD and CAVE)
Outcomes	Reporting at least one environmental attribute investigated in relation to walking/cycling Objective and subjective outcome measurement Additional outcome(s): health-related quality of life	Optimizing VR methodology/software
Study design	Experimental designs	

Articles were included if they reported at least one geographical environment attribute investigated in relation to active mobility and related attitudes, including both objective and subjective outcome measurements. Studies were excluded from the analysis if they: (1) represented an indoor environment; (2) targeted disease-specific population groups or used VR for rehabilitation/therapy; (3) were related to driving simulation; (4) focused on non-relevant disciplines (e.g., military training, emergency evacuation, and education); (5) where participants had no physical movement (redirected using joystick, torso, or gaze); (6) addressed participants younger than 18 years as they may interact less independently with their geographic environments and exhibit different (VR) environment-active mobility behavior relationships than adults; and (7) optimized the VR methodology or software.

### Selection process

**Fig. 1:**
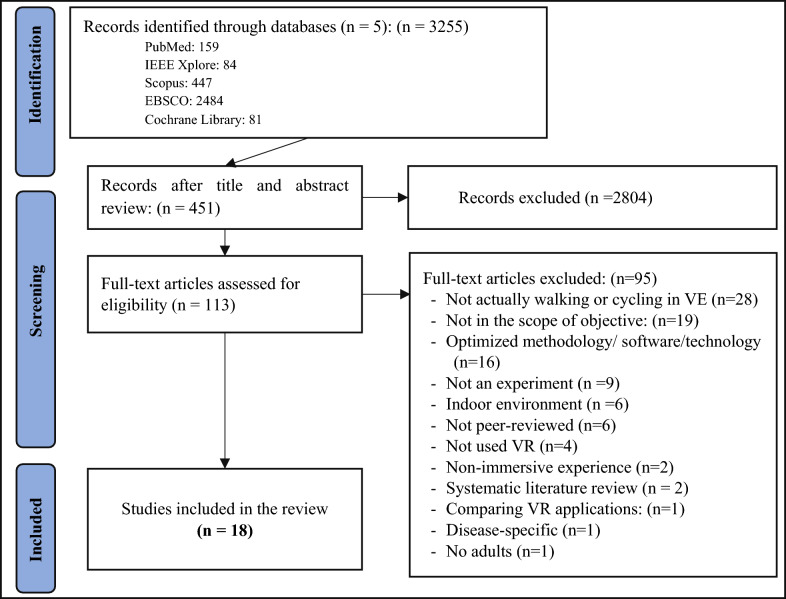
PRISMA flowchart

The selection process consisted of three phases including identification, screening, and selection process, as Fig. 1 presents the PRISMA flowchart. The literature search was conducted by the first reviewer (MG) and yielded 3255 records. In the first phase, titles were screened, and 2804 articles were excluded based on inclusion and exclusion criteria. In case of doubt, papers were included in the abstract review phase. In the second phase, the selected articles underwent abstract review by three independent reviewers (MG, CP, and MD). Any disagreement was resolved through team discussion with a fourth reviewer (RM). Finally, the remaining records were fully reviewed, and another 95 articles were excluded, yielding 18 articles.

### Data extraction

A ten-item extraction table was used to extract data from included studies under the following headings: (1) general information; (2) population characteristics; (3) study design; (4) sessions characteristics, (5) Active mobility measurements (6) environment measurements; (7) VR measurements; (8) statistical analysis; (9) results, and (10) conclusion. Appendix 1: Table 2 provides further details.

### Quality assessment

To assess the risk of bias, the QUALSYST quality assessment tool from the "Standard Quality Assessment Criteria for Evaluating Primary Research Papers from a Variety of Fields" was used [44]. Based on the study designs of the included research, only the checklist designed for assessing the quality of quantitative studies was utilized (Appendix 1:Table 3). Fourteen items were scored based on the degree to which the specific criteria were met (“yes” = 2, “partial” = 1, “no” = 0). To calculate the summary score, items that were not applicable to a particular study design were marked as 'n/a' and excluded. For each paper, a summary score was calculated by summing the total score obtained across relevant items and dividing by the total possible score. To maintain quality control, a quality assurance process allowed for cross-checking of the quality assessments, and any discrepancies were addressed and resolved through discussions.

## Results

### Study characteristics

Table 2 provides a detailed overview of all the study characteristics that were included. Among the 18 studies included, 4 were conducted in the United States [26, 45–47], 9 in Europe (i.e., United Kingdom [48], France [40, 49], the Netherlands [21], Norway [50], Italy [51], Czech Republic [52], Greece [27], and Germany [53]), and 4 in Asia (i.e., Israel [4] and Singapore [24, 54, 55]). The remaining study did not mention where the study was carried out [25].

**Table 2:** Overview of study characteristics

Authors	Population characteristics	Study design		Sessions		Active mobility measurements			Environment		VR measurements	Result	Quality score
	Sample size (number, gender, age)	R/NR^a^	WS/BS^b^	No^c^	Session characteristics	W/C^d^	Objective measurements of active mobility	Self-reported measurements of active mobility	N/B/S^e^	Environmental attribute	The degree of Immersion/Reality	Impacts of geographical environments associated with active mobility on people	
[45]	48 students, 60% women	R	WS	1	1. Instructions 2. Exposure to 8 scenarios and in-between surveys	W	–	Environmental perception	N	Spatial enclosure perception using the concepts of visual and locomotive permeability (low, medium, and high enclosed)	–	1. Perceived park safety relies on vegetation-based spatial enclosure levels and the presence of non-threatening people and paths (social and environmental cues) 2. Safety perception decreases with increased spatial enclosure: low is safest, medium is next, and high is least safe	0.67
[49]	16 students and staff, 25% women, M-age = 24.9	NA	WS	2	1. Two training trials 2. Four trials in each condition: real vs. virtual environment	W	–	–	S	Crowd density similar to real environment	–	1. Smaller gaze coverage in VE 2. Accuracy of reproducing features in the VE impacted participants' gaze behavior 3. Gaze fixations centered in the field of view, emphasizing participants' focus on the central task in both RE and VE	0.67
	18 student and staff, 22% women, M-age = 25.5	R	WS	1	1. Familiarization 2. Four trials for each condition		–	–		Crowd density in 6 conditions: from low (2 people) to high (24 people)	–	1. More focused participants' gaze in high-density conditions 2. Density affects eye movements not gaze duration	
[21]	86 student and staff, 31.4% women, IVR: m-age = 28.2	R	BS	1	1. Introductory phase 2. Four random cycling segments out of 8 scenarios, 3. Within VE survey and after exposure survey	C	–	Environmental perception (stated preference)	B	1. Greenness 2. Path width and path separation 3. Traffic volume	ITC-Sense of Presence Inventory questionnaire	1. Greenness: The most important attribute for aesthetics and enjoyment, with the greatest impact on overall attractiveness 2. Cycle Lane Width: Key for safety perception and the second most significant attribute 3. Traffic Volume: Has the lowest impacts	0.88
[4]	4 colleagues, 75% women, 30–45	NR	WS	2	1. IVE exposure 2. First survey 3. IVE exposure 4. Second survey	W	1.Walking durations 2.Walking distance 3.Speed 4.Number of steps 5.Cadence (steps/min) 6.Step regularity 7.Step symmetry, 8.EDA^f^, 9.HR^g^	Affective responses (enjoyment)	B	1. Greenness 2. Parked car	NA	1. Green environment positively influenced enjoyment 2. Participants showed varying attention to details, with most focusing on a yellow car	0.67
[55]	48 students, university employees, and general public members, M-age = 26.5	NA	WS	1	1. Familiarization and trial session 2.Exposure to 6 IVE	C	1. Cycling speed 2. Acceleration 3. α wave amplitude using EEG^h^	–	B	1. Presence of a car at the junction 2. Constant: Cycling on painted lane, separated from pedestrian zone, and segregated from the road traffic by a grass strip	Sounds according to environment's characteristics	1. Approaching junctions with the presence of cars increases cyclists' likelihood to slow down and brake, leading to a perceived increase in risk 2. Higher risk induces more braking	0.63
[50]	26, 46% Women, M-age = 26+_ 8 years	R	WS	1	1. Three experimental conditions: nature walk, sitting-IVE, and treadmill-IVE 2. in-between condition questionnaires	W	1.Walking speed 2.HR 3.PAAS (physical activity affect scale)	1. Affective responses (enjoyment and affect) 3.Environmental perceptions (perceived environmental restorativeness, using Ratings of perceived exertions)	N	Blue environment (paved trail along a large river) Built elements (buildings & football field)	Sound: footsteps and people's voices	1. IVEs had comparable restorative and physically engaging qualities but elicited more negative emotions than real nature walks 2. Real nature walks positively impacted participants' emotions by reducing fatigue and negative feelings, leading to enjoyment 3. Significantly less enjoyment reported in the treadmill condition than in outdoor walking 4. No significant differences in HR and walking speed observed between outdoor walking and treadmill walking	0.67
[54]	Pilot: 12 students, 50% women	R	WS	2	1.VR exposure 2. Post-test survey	W	–	Environmental perception (complexity, enclosure, and interest)	N vs B	Nature connectedness	Igroup presence questionnaire (IPQ)	No differences in spatial presence, involvement, realism, 'being there' sensation, perceived complexity, enclosure, and interest between nature and urban environments	0.83
	30 students, 70% women, M-age: 20.5	R		2	1. Pre-test survey 2.VR task 3.Post-test survey		Electrical activity of the heart using ECG^i^	Affective responses (Positive and negative affect (using PANAS))			Full auditory and visual immersion	1- Nature VE: improved emotional state, reduced stress, increased relaxation, and enhanced sense of nature connectedness 2. Urban VE: positive effects decreased significantly, and increased stress	
[51]	36 students, 50% women, 18–37 (M-age = 22.3)	R	Mixed	1	1. Familiarization 2. Exposure to 4 conditions 3. Interview, and measuring length of participants arms	W	Distance from nearby virtual stimuli	Environmental perception (Peripersonal and interpersonal space)	S	Presence of others (social environment): Human, anthropomorphic robot, and a cylinder) in 4 condition: 1. Passive-comfort distance, 2. Active-comfort distance, 3.Passive-reachability, 4. Active-reachability	–	Reachability and comfort distance influenced by gender (larger in females), approach condition (larger in passive), and type of virtual stimuli (larger from cylinder, smaller from virtual females)	0.75
[53]	51: 60% students, 40% heterogeneous professions and age group, 65% women, 15–77, M-age = 31.94	R	Mixed	1	1. Baseline condition: Lake sceneries in the woods, accompanied by sounds 2. Four Conditions followed by a questionnaire 3. Baseline condition	W	HR and EDA	Affective responses (PANAS)	N	1. Soundscape in 4 conditions: silence, a nature soundscape, and music of positive or negative valence 2. Time of day (daytime vs. nighttime)	Presence questionnaire (IPQ)	1. Sound conditions strongly influenced positive feelings; silence was different 2. Music generated higher emotional reactions than the soundscape 3. Sound reproduction per se leads to higher Presence ratings	0.75
[25]	80 students, 28% women, 19–37 (M-age = 22.51)	NA	BS	1	1.Demographics 2.Short walk trial 3.Ten virtual crossing tasks 4.Experiment feedback	W	1. Walking speed 2.Walking distance 3.Time, 4.Distance from nearby pedestrians 5.Direction 6.Smoothness	Affective responses (narrative feedback on their movement behavior)	S	Virtual crowd (medium density)	Sounds of cities	Different crossing behavior with simulated crowd: Participants moved slower, followed longer paths, performed less-smooth motions, and allowed more distance between themselves and nearby pedestrians	0.67
[26]	80 university setting, 28% women, M-age = 22.51	NR	BS	1	1. Instructions 2. Exposure to 8 conditions in 3 pairs of conditions 3. Experiment feedback	W	1. Walking speed 2. deviation 3. Walking distance	–	S	Virtual crowd: 1. Density 2. Walking Speed 3. Direction	Sounds of cities	Significant impact on participant's movement behavior within virtual crowd: High density was associated with low speed, diagonal direction situations, and longer trajectory length Low density was associated with ↑ speed, straight-direction crowd scenarios, shorter trajectory length	0.65
[27]	42students, 21% women, 19–27 (M-age = 21.55)	R	WS	1	1. Demographics 2. Familiarization 3. Crossing task followed by questionnaire for 6 conditions 4. Voluntary feedback	W	1. Walking speed, 2. distance, 3.duration, 4.smoothness, 5.deviation, 6.direction	Affective responses (emotional reactivity to the tactile feedback, and associated with crowd interaction) Environmental perception (participants’ sensation of colliding with the virtual bodies)	S	Navigating through a highly dense virtual crowd walking toward the opposite side of the street in 6 conditions: 1. No TF^j^ (Baseline), 2. Side TF, 3.Back TF, 4. Front TF, 5. Accurate TF, 6.Random TF	Sounds of cities	Significant: speed of the participants (greatest impact: front TF), length and deviation measurements (side and random TF), duration (front and back TF) Not significant: smoothness and direction measurement	0.79
[40]	E1^k^:36 students, 80% women, M-age = 21.53	R	WS	1	1.Sociodemographics 2. Route learning 3. 4 trials (2uphills 2 downhills) in each: route learning, questionnaire, interfering task, landmark recognition test, route decision test, landmark position retrieval test, and pause 4. Users' estimation of travel time and distance followed by questionnaires	W	–	Environmental perception (spatial span test, users' estimation of distance and travel time)	B	1. Various degrees of inclination (M-slope:4.7%/20 m) (uphill or downhill) 3. 32 different landmarks of various sizes, colors, and functions	Presence questionnaire (Witmer et al. 2005)	1. Walking uphill routes led to distance underestimation compared to downhill routes 2. Significant findings: Slope direction significantly predicted performance in route decision, distance accuracy, inter-landmark angular accuracy, landmark positions, and route perspective preference 3. Not significant: Slope direction did not predict implicit walking speed estimations for distance and time	0.92
	E2: 45 students, 75% women, M-age = 20.57	R	WS	1					B	Same but participants wore loaded ankle weights		The distance underestimation effect in uphill routes (E1) disappeared	
[24]	150 students and people from Land Transport Authority of Singapore, 52% women, M-age = 27.0	R	Mixed	1	1. Questionnaire 2. Cycling 5 different environments followed by questionnaires	C	–	Environmental perception (safety) Affective response (willingness to bicycle)	B	Car traffic volume: low vs. high Bicycling environment: 1. Sidewalk next to pedestrians, 2. Painted bicycle path on the sidewalk, 3. Painted bicycle path on the road, 4. Roadside next to vehicles, 5. Segregated bicycle path	–	1. Ranking safety and willingness to bicycle: Segregated bicycle path ranked highest, followed by painted path on the road, painted path on the sidewalk, roadside bicycling, and bicycling on the sidewalk. Segregated paths were deemed the safest 2. All participants would consider bicycling for trips under 10 min, regardless of infrastructure or traffic volume 3. Traffic volume did not impact bicyclists' perceived level of safety	0.96
[52]	126, M-age: C^l^1:M = 25.6, C2:M = 25.7, C3:M = 25, C4:M = 22.8 C5:M = 22.9 C6:M = 28	R	BS	1	1. Six different experimental conditions, including uni- and bimodal stimuli (auditory and visual) 2.Questionnaire	W	–	–	N	Soundscape in 6 conditions: 1) Visual only, 2) Visual with footstep sounds, 3) Visual with full sound (static and 3D sound), 4) Visual with fully sequenced sound, 5) Visual with sound + 3D sound, and 6) Visual with music	Sound of nature, presence questionnaire	1. Combination of soundscapes, 3D sound, and auditory rendering of one’s own motion in VE induces a higher degree of motion 2. Condition 6 (music) induces the least movement, even less than the Visual-only condition	0.42
[48]	18, 44% women, 20–45 (M-age = 30.06)	R	WS	1	1. Demographics 2. Baseline: Real-world tasks followed by a virtual replica 3. 8 VE (4 indoor and 4 outdoor) followed by a questionnaire and an interview 4. Task repetition, starting with a virtual replica followed by the real world	W	Deviation Deviation. Area Curvature Length Time Speed	Environmental perception questions	N	1. Greenness (Grassy area with trees and rocks) 2. Specified walking path 3. Blue environment (shallow pond, ice)	Sounds according to environment's characteristics	1. Alteration in participants' trajectories in the presence of surfaces representing higher walking difficulty (water instead of grass) 2. No significant trajectory changes when virtual objects appeared strange but not confusing 3. User behavior is influenced by real-life experiences and expectations of how the virtual world responds to their actions	0.83
[47]	106 students, 53% women	R	Mixed	1	1. Familiarization 2. 1 practice trial + 20 test trials 3. Demographics	W	Road crossing duration Gaps number and size	–	B	AHS^m^ treatment conditions varied in color (white or red) and timing of an icon projected on the roadway as an AHS vehicle approached	Traffic sound	1. Pedestrians exhibit similar road-crossing behavior during day and night, potentially contributing to high nighttime fatalities 2. Interaction with AHS-equipped vehicles may encourage safer crossing behavior 3. Pedestrians were willing to cross roads with tight gaps at night 4. Warnings can appear 4 to 4.5 s before the vehicle reaches the crossing line	0.81
[46]	E1:10	R	WS		1.Series of practice and test trials 2. E1: 96 trials(8 conditions)	W	Walking direction Walking speed	–	S	Virtual crowd: 24 humans, random directions ± 10° or ± 20° left or right	Frame rate of 30–60 fps	Participants are attracted to the crowd’s mean heading, regardless of the amount of crowd noise	0.69
	E2: 12	R	WS		E2: 96trials (12 conditions)					Splitting virtual crowd of 36 into 2 groups with distinct heading directions and varying proportion	Frame rate of 30–60 fps	Participants walked in the mean heading direction of the crowd in all conditions, despite the largest angular difference between groups	
	E3:12	R	WS		E3:120 trials (15 conditions)					Virtual crowd: 48 individuals with random directions (180° range), followed by a subgroup of neighbors changing direction by ± 20° after a few seconds	Frame rate of 45–90 fps	1. The mean final heading gradually shifted with crowd mean as subgroup percentage increased from 0 to 100%: 2. Subgroup attraction is based on mean walking deviation impact, not coherence	

^a^Randomized or Non-randomized

^b^Control group: within subject-comparison or between-subject

^c^Number of sessions per experiment

^d^Walking or Cycling

^e^Nature (N), Built environment (B), Social environment (S)

^f^EDA—electrodermal activity

^g^Heart rate

^h^Electroencephalography

^i^Cardiac Electrical Activity

^j^Tactile feedback

^k^Experiment

^l^Condition

^m^Adaptive Headlight Systems

All studies were experimental, 9 of them used within-subjects, and 4 used a between-subjects study design. Four studies used a mixed-study design [24, 47, 51, 53].

### Participants

Participants' sample sizes ranged from 4 [4] to 150 adults [24] with ages ranging from 18 to over 65 years. In terms of gender, 15 studies included both men and women, and 3 studies did not report gender information [46, 52, 55]. Overall, 78% (n = 14) of the study participants were either students, university employees or colleagues while the remaining studies did not provide information about participants ‘occupations. Only two studies included the general population [55] and specific target population (i.e., Land Transport Authority) [24]. No studies reported participants’ ethnicity.

#### Number of withdrawals, exclusions, lost to follow-up and reasons

Six studies reported participant withdrawals, exclusions, or failures to follow up during their experiments [21, 40, 47, 49, 54, 55], with only one study reporting exclusions due to symptoms of cybersickness caused by VE [40].

The main reasons for participant exclusion during the data analysis phase were incorrect eye-tracking calibration [49], non-qualified data driven from an electrocardiogram (ECG) [54], and a combination of technical issues and participants failing to adhere to the study instructions [47].

### Risk of bias

The quality scores among the studies ranged from 42 to 96% (with 0% being the worst and 100% being the best), as presented in Table 2. Item 6 ‘If interventional and blinding of investigators was possible, was it reported?’, and item 7 ‘If interventional and blinding of subjects was possible, was it reported?’ were not applicable to these studies. Notably, item 12 ‘Controlled for confounding?’ appeared to be the most frequently missed among studies. Interestingly, studies had an average score of 73%, indicating an average good quality.

### Synthesis of results

#### Geographical environment attributes

Among the 18 studies included, 33% (N = 6) were carried out in a built environment [4, 21, 24, 40, 47, 55], 28% (N = 5) were conducted in nature [45, 48, 50, 52, 53], and 33% (N = 6) explored the social environment [25–27, 46, 49, 51]. In addition, one study compared nature with the built environment [54]. Geographical environment attributes can be categorized into static and dynamic. Static attributes remain constant over time, while dynamic attributes are non-stationary factors that might change or move in the VE (i.e., the presence of people, cyclists, cars, and their interactions). Table 3 references all the geographical environment attributes investigated.

**Table 3:** Geographical environment attributes

	Geographical environments attributes	Walking/Cycling	References
Static	Greenness	W	[4, 45, 48, 54]
		C	[21]
	Blue environment	W	[48, 50]
	Built elements (i.e., buildings, football field, junction)	W	[50]
		C	[55]
	Various directions of street inclination	W	[40]
	Landmarks	W	[40]
	Path width	C	[21]
	Path separation	C	[21, 24, 55]
	Parked car	W	[4]
	Time of the day	W	[53]
Dynamic	Crowd density	W	[25–27, 46, 49, 51]
	Traffic volume/condition/speed/direction (Pedestrian, cyclists, and cars)	C	[21, 24, 55]
	Soundscape	W	[52, 53]
	Crossing gap (Car’s Adaptive Headlight Systems)	W	[47]

##### Walking environmental correlates

Static attributes investigated in relation to walking comprise greenness/vegetation, blue environment, built elements, street inclinations, parked car, time of the day, and landmarks. Dynamic attributes include crowd density, soundscape, and car’s adaptive headlight systems (AHS).

Greenness was measured as the presence of greenery (vs. absence) in terms of trees along the street [4], grassy areas with trees [48], and spatial enclosures shaped by vegetation, including trees, bushes, and grass [45]. These green attributes were explored in relation to aesthetics [4, 21], stress [54], well-being, and perceived safety [45], and nature connectedness (i.e., one’s subjective sense of feeling connected to the natural world)[54].

Blue environment was investigated as a walk along a river in combination with built elements [50], and the presence of a shallow pond (vs. absence) to measure people’s movement alterations [48].

The impact of landmarks on perceived walking distance at various street inclinations was examined in relation with route decisions and spatial memory [40]. Additionally, pupil fixation on a parked car was investigated using eye tracking [4].

The influence of time of the day (daytime vs. nighttime) on the positive and negative affects experienced during the walk was investigated [53].

Social environment was studied in five distinct ways:

1) Observing individuals walking within a virtual crowd with varying densities (i.e., from 1.5 pedestrians per square meter to 24 in the VE) [25, 49].

2) Investigating impacts of crowd density (low: 1 pedestrian vs. high: 2.5 pedestrians per square meter), walking speed (low: 1.2 m/s vs. high: 3.8 m/s), and walking direction (straight vs. diagonal) on movement behaviors [26].

3) Assessing the impacts of tactile feedback (i.e., a sensory experience within a crowd), on movement behavior [27].

4) Investigating the effects of crowds with diverging motions and dividing the crowd into distinct subgroups, each with different proportions, influencing participants' path choices [46].

5) Exploring reachability and comfort distance judgements toward humans and objects while standing still (passive) or walking toward stimuli (active) [51].

Soundscape mimicking the presence of pedestrians, cyclists, and cars as well as their interactions, was investigated in various aspects including presence vs. absence [52, 53], auditory feedback (footstep sounds) [52], static vs. 3D sound [52], and music [52, 53].

Finally, presence or absence of a car's AHS was explored in terms of the color (white vs. red) and the timing of an icon projected onto the road. This icon was part of the dynamic attributes of the environment while participants crossed a road [47].

##### Cycling environmental correlates

Geographical environment attributes examined in relation to cycling behaviors included cycling path width and separation [21, 24, 55], greenness [21], and traffic volume [21, 24, 55]. Path separation conditions included sidewalk next to pedestrians, painted bicycle path on the sidewalk, painted bicycle path on the road, roadside next to vehicles, and segregated bicycle path [24, 55]. Furthermore, path width (wide vs. narrow) was investigated in combination with path separation (well-separated vs. poorly-separated) [21].

Presence (vs. absence) of greenness was explored in relation to aesthetics using a stated preference conjoint experiment [21]. Additionally, in terms of traffic volume, car traffic volumes (high vs. low) were assessed in relation to perceived levels of safety [24], and pedestrian and cyclist traffic volumes (high vs. low) were investigated in relation to enjoyment [21]. Moreover, cyclists' behaviors at street junctions were examined in relation to the presence (or absence) of car traffic [55].

#### VR measurements

Most experiments used HMD (N = 17), with only one study employing CAVE [47]. For detailed information regarding the different models of HMD or VR glasses, and CAVE setups, refer to Appendix 1: Table 4.

##### User’s natural interaction with virtual environment (VE)

User natural interaction with the VE refers to an individual's intuitive engagement with VE that simulates real-world interactions [56]. This is crucial for understanding the degree of realism and effectiveness of the virtual experience. Four interaction dimensions were introduced to describe participants' VR locomotion experiences:

1. Immersion: how the technique (e.g., walking in the place) supports users’ attention in the virtual task and environment and alters their sense of space, time and self.
2. Ease-of-use and mastering: how operating the technique (e.g., using a controller) can be learned and can enable efficient navigation.
3. Competence and sense of effectiveness: how the technique can assist the users in accomplishing their goals and tasks.
4. Psychophysical discomfort: if the technique causes fear, motion-sickness, and tiredness [57].

Overall, 67% (n = 12) of studies have reported on different aspects of users’ level of natural interaction with VR [4, 21, 24–27, 40, 50–54]. Five of them indicated low levels of natural interaction due to challenges in adjusting to the VE [4], poor graphic quality and movement lag [50]. Three of them reported low levels of interaction in specific conditions; for instance, levels of user natural interaction were lower among participants with prior experience with VR [4], in silent experimental conditions [53], or within an uphill street slope condition that significantly caused difficulty in path recall and distance estimation [40].

##### The degree of immersion/presence

Immersion and presence are two related fundamental concepts of VR. Immersion refers to the psychological state experienced by an individual perceiving themselves as deeply engaged in a VE [58]. Presence, in the context of immersion, is the perceptual and psychological state of profound involvement and absorption in a VE [59].

In total, 50% of studies reported immersion indicators, such as sound (N = 8) and frame rate (N = 1) (Table 4). Additionally, 4 studies assessed immersion and presence using questionnaires, including Swedish Viewer-User Presence questionnaire [52], adapted questions from Slater et al. presence questionnaire [27], the revised version of presence questionnaire by Witmer et al. [40, 60], and ITC-Sense of Presence Inventory [21], [61]).

**Table 4:** Immersion indicators

Immersion indicators	Types	References
Sound: (n = 8)	Sounds of cities (e.g. traffic and noise)	[25–27, 47]
	Sounds of nature (e.g. birds and waterfalls)	[52]
	Voices of other people passing by	[50]
	Unspecified	[48, 55]
Frame rate	Higher frame rate means more images are displayed, resulting in a smoother VR experience	[46]

Overall, experiments utilizing HMDs reported high levels of immersion [21, 25–27, 46, 54]. The sense of presence was found to be highly correlated with sound information and localization [52]. Notably, the voices of other people passing by [50] seemed to evoke the feeling of “being there”. Additionally, the frame rate, which refers to the number of individual images displayed per second, affects the realism and engagement of the user experience [46].

##### Length of exposure to VEs

Table 5 summarizes the length of exposure to VEs, categorized into less than 10 min, between 10 and 20 min, and more than 20 min. Importantly, 9 studies did not report the length of exposure to VE [25–27, 40, 46–48, 52, 55]. One study reported the length of exposure in terms of distance rather than duration (i.e., 2 times of 70 m walk) [4].

**Table 5:** Length of exposure to VEs

Categories of length of exposure	References
Less or equal to 10 min (n = 3)	[21]: 10 min; [54]: 8 min (pilot); [24]: 5.75 min
More than 10 to 20 min per participant (n = 4)	[50]: 20 min; [54]:14 min; [45]: 12 min; [49]: 15miutens
More than 20 min (n = 1)	[51]: 24 min; [53]: 28 min

##### Real and virtual comparison

Two studies compared active mobility behaviors in real vs. virtual environments [49, 50]. Berton et al. investigated biases introduced by VR in visual activity during walking [49]. To examine whether green exercise in Immersive Virtual Environment (IVE) elicits psychological responses similar to those experienced in natural environments, Calogiuri et al. compared outdoor walking in a natural environment, sedentary exposure to an IVE, and treadmill walking while watching the same IVE [50]. These studies demonstrate both the potential and the limitations of utilizing VR in replicating real-world conditions, emphasizing the need for controlled experimental design and cautious interpretation of results aligned with VR-related experimental setting limitations.

##### Cybersickness

Cybersickness, simulator sickness, or motion sickness, is a challenge for VR experiments, and results in headaches, dizziness, eye strain, disorientation, and nausea [62]. Among the 7 studies that reported the occurrence of cybersinkess (39%), 2 reported symptoms of cybersickness [40, 50], while 5 indicated no symptoms of cybersickness [24–27, 54].

##### Motion in VR

The embodied experience of VE is further linked to motion techniques. Studies were carried out using various techniques such as a walking simulator [4, 40, 50, 53], cycling simulator [21, 24, 55], CAVE environment [47], or controlled laboratory setting, to conduct the VR experiments [25–27, 45, 46, 48, 49, 51, 52, 54]. Table 6 reports information on types of motion techniques.

**Table 6:** Motion techniques in VR

Motion techniques	Type	References
Walking simulator	Omnidirectional treadmill	[4, 40]
	Manually driven treadmill	[50]
	Fitness-training treadmill	[53]
Cycling simulator	Standard Dutch bicycle fixed to Elite RealAxiom Wired (an electromagnetic trainer)	[21]
	Instrumented bicycle	[55]
	Instrumented bicycle with series of rotation sensors	[24]
CAVE environment		[47]
Controlled laboratory setting	Ranging from 7.2 m2 [51]–168 m2 [46]	[25–27, 45, 46, 48, 49, 51, 52, 54]

##### Complementary technologies combined with VR

VR has the potential to integrate with other technologies to provide complementary information on participants' momentary responses to the VE. For instance, sound simulation systems can be employed to enhance the realism of user experiences. Table 7 presents these technologies, categorizing them into input and output devices.

**Table 7:** Complementary technologies combined with VR

Input/output device	Technology/method	Device type	References
Input devices	Eye-tracking	Built-in eye tracking	[4, 49]
	Motion tracking	Xsens inertial motion capture system	[25–27]
		Polhemus IsoTrak II3 tracker	[52]
		Empatica E4	[4, 53]
		Positional tracking in HMD	[24, 27]
		InertiaCube3 and Precision Position Tracker, PPT-H4	[51]
		Odyssey’s inside-out tracking system and IS-900 inertial/ultrasonic tracking system	[46]
		OptiTrack motion capture system	[47]
		Garmin Forerunner 310 XT	[50]
	Hand tracking	Data Glove	[48, 51]
Output devices	Haptic feedback device	Haptics tactile vest	[27]
	Sound mimicking headsets	Shark Zone H10 Gaming Headset (Sharkoon Technologies GmbH, Linden, Germany)	[25]
		Sennheiser HD 201 headset	[50]
		Dynaudio BM5A speakers	[52]

##### VR experience of participants

Participants with prior VR experience might behave differently from those without. Only 6 studies (33%) reported participants' previous experience with VR [4, 21, 25, 46, 48, 54].

#### Measurement of active mobility behaviors

Among 18 selected articles, 15 focused on walking [4, 25–27, 40, 45–54], and 3 on cycling [21, 24, 55]. Walking-related studies were investigating outcomes such as road crossing, crowd walking, walking behavior, perceptions, physical engagement, and wayfinding behavior. Cycling behavior and perceptions were investigated in cycling-related studies.

Four studies relied exclusively on objective measures of walking and cycling behaviors [26, 46, 47, 55], 4 studies exclusively used self-reported measures [21, 24, 40, 45], and 8 studies used both self-reports and objective measures [4, 25, 27, 48, 50, 51, 53, 54]. Finally, two studies did not assess any objectively measured or self-reported active mobility attributes [49, 52]. Table 8 presents the objective measures of walking and cycling.

**Table 8:** Objective measurement of walking and cycling

Objective measurement	Measures	References
Walking/Cycling characteristics	Walking: speed, distance, duration, direction, deviation, distance from nearby pedestrians, number of steps, cadence (steps/min), step regularity, and step symmetry	[4, 25–27, 46–48, 50, 51]
	Cycling: speed, acceleration	[55]
Wearable devices/Sensor-based measurements	HR, HRV, EDA, SC, brain activity using EEG, Heart electrical activity using ECG for measuring Cardio-vascular activity, BVP, gait sensors	[4, 50, 53–55]

HR: Heart Rate; HRV: Heart Rate Variability; EDA: Electrodermal activity; SC: Skin conductance level, EEG: Electroencephalography; ECG: Cardiac Electrical Activity; BVP: Blood Volume Pulse

Twelve studies measured attributes of walking and cycling using self-reported measurements. These attributes are categorized in Table 9, into two main categories: environmental perception and affective responses.

**Table 9:** Self-reported measurements of walking and cycling

Self-reported measurements	References
Environmental perception (e.g., safety perception, space perception)	[21, 24, 27, 40, 45, 48, 50, 51, 54]
Affective responses (e.g., positive and negative affect, enjoyment)	[4, 24, 25, 27, 50, 53, 54]

## Discussion

This review aimed to summarize the existing literature on the attributes of geographical environments in relation to walking and cycling behaviors using VR, as well as to identify gaps in the literature for future investigations. The results from 18 peer-reviewed papers highlighted the positive impacts of environmental attributes such as greenness and pathway design on relaxation and stress, alongside their effects on movement behavior. Crowd density and traffic have been associated with behavioral adjustments, such as slower walking speed and increased braking. However, a major gap revolves around the notable need for broader research exploring a more diverse array of these attributes—using the 5Ds, as well as a wider including of both static and dynamic attributes, as their complex interplay may significantly influence walking and cycling behaviors. We discuss the identified environmental attributes and their impacts, then highlight the identified gaps. Building on these observations and identified gaps, this discussion reviews VR’s potential and limitations in such studies and concludes with future research directions.

### Virtual geographic environments and their impacts on walking and cycling behaviors: toward more diversity and complexity

The most common static attributes were greenness, blue environments, and path width and separation, while dynamic attributes that recurred most often were crowd density, traffic, and soundscape. Greenness exhibited associations with improved emotional state, reduced stress, increased relaxation, and enhanced sense of nature connectedness [54], further confirming that exposure to a green environment promotes positive emotions, as reflected in the affective responses [4, 21, 45, 54]. Furthermore, greenness ranked highest for aesthetics and enjoyment [4, 21], and when it formed open spaces (low enclosure), it was positively linked to perceived safety [45]. Similarly, walking in a blue environment correlated positively with restorative and physical engagement [50].

In comparing natural and built environments, no differences were observed between the nature VE and urban VE (i.e., path between buildings in a downtown area) in terms of spatial presence, realism, enclosure, perceived complexity, or interest [54]. However, physiological measurements revealed that urban VE decreased positive emotions and increased stress, while nature VE was associated with a significant decrease in heart rate (HR) and HR variability, suggesting greater relaxation and reduced stress [54]. Nevertheless, in another study, higher electrodermal activity and HR levels in the green route were attributed to increased sweating while walking rather than indicating increased emotional arousal [4]. Therefore, reproducing real-world walking and cycling conditions in experimental settings requires careful consideration, as stress measures and sensor data may be subject to biases introduced by walking and/or cycling.

Path width and separation, so far only investigated in cycling-related research, heavily influence traffic safety perception [21, 24]. Segregated bicycle paths were considered the ideal option, followed by painted bicycle lanes on roads or sidewalks [24]. Interestingly, duration and purpose of the trip were factors influencing willingness to bicycle [24].

In studies exploring social environments, walking within virtual crowds triggered various behavioral responses. Higher crowd density is correlated with slower movement, longer trajectories, less smooth motions, and greater distances from nearby pedestrians [25, 54]. When the virtual crowd's heading varied randomly or split into groups, participants often moved toward the crowd's average heading [46]. Tactile feedback presence influenced participants' speed, trajectory, and walking duration [27]. Furthermore, the effects of reachability and comfort distance varied by gender (larger in females), approach condition (larger effects in passive condition), and type of virtual stimuli, leading participants to prefer more distance in conditions that they could not control (passive) and with virtual female avatars [51].

Soundscapes were generally associated with positive feelings and resulted in higher presence ratings [52, 53], while music generated higher emotional reactions than soundscape [53]. Furthermore, combining 3D sound and auditory rendering of one's own motion in VE induced a greater sense of motion [52].

Traffic, which has only been studied in cycling-related research, had a low impact on cyclists’ environmental perceptions [21, 24] and enjoyment [21], but in street junctions induced more braking and reduced speed [55]. Additionally, findings indicated that higher amplitude of alpha brainwaves—derived from the electroencephalography—is associated with a higher perceived risk of collision, thereby increasing the probability of braking [55].

Other street-related attributes influenced people's active mobility behavior. Walking on an inclined street, the slope direction (uphill vs. downhill) significantly affects participants' spatial cognition [40]. Furthermore, road crossing duration was equivalent during day and night, while participants tended to cross roads with tighter gaps at night. [47].

### Bridging gaps with VR: toward more diversity and complexity using the 5D's

Overall, there has been limited exploration of geographical environment attributes using VR, while VR technology does open up new opportunities to experiment with a wider variety of environmental correlates of walking and cycling. This calls for a broader investigation into diverse geographical environment attributes using the 5Ds [36].

Studies predominantly focused on the "Design" dimension of the 5Ds, assessing green spaces [4, 21, 45, 48, 54], path width [21, 24, 55], and street inclinations [40]. "Density" has also been explored in terms of population [25–27, 46, 49, 51] and traffic [21, 24, 55], while "Destination" was investigated only in one study [40] in terms of spatial learning. Yet, dimensions such as 'Diversity' in land use, exploring mono-functional vs. multi-functional environments, and "Density" in terms of building, population, or traffic, need further investigations. Additionally, a more detailed investigation of "Design" attributes such as street and sidewalk connectivity, characteristics of streets and related infrastructures, sidewalks, bike lanes, and crossings are required. Similarly, the "Destination" and "Distance" aspects—focusing on the distance between key locations and proximity to transit options—could provide valuable insights into active mobility correlates when explored in virtual scenarios.

Additionally, it is crucial to consider a combination of static and dynamic attributes such as car traffic, and population density, as they interact in complex ways to influence active mobility behaviors. For instance, density may not promote active mobility per se, but it serves as a proxy for other environmental factors, such as demographics, access to local destinations and public transport, and connected street networks, which directly influence individuals' choices of transportation and thus active mobility [63].

### VE: realism, reliability, but still limited knowledge

VR technology is an effective tool for assessing perceptions and attitudes of pedestrians and cyclists, providing a safe, convenient, and realistic representation of potentially dangerous/risky situations [7, 23, 24, 45]. HMDs were more widely used than CAVEs, possibly due to the greater immersion, affordability and convenience of set-up [4, 64]. Immersion and presence were significant factors in creating complex aspects of real-life environments, and highly correlated with the inclusion of sound in VEs. User natural interaction was associated with graphic quality [50], prior VR experience [4], the presence of auditory stimuli [53], and the complexity of the VE [40].

#### VR’s strength in experimental studies

VR's unique strength to test dynamic attributes in combination with static ones [21], makes it a powerful tool for exploring how geographical environment attributes may interact to promote or hinder active mobility. VR offers realistic and dynamic environments, provoking complex behaviors similar to the real world. Researchers can precisely manipulate environmental conditions with high levels of experimental control [4, 21, 25, 51, 54], replicating specific scenarios and testing various environmental attributes to assess their independent and/or potentially interactive impacts on walking/cycling behaviors [21, 25, 45, 55]. It simultaneously upholds high experimental validity by ensuring consistent conditions across participants, minimizing confounding variables, and enhancing ecological validity, which refers to the extent to which the research task approximates a real-life situation [4, 45, 51]. Furthermore, VR experiments are highly enjoyable and engaging for participants due to their novelty and appeal [21]. This attractiveness facilitates the recruitment of participants, but needs further exploration for how it influences dedication to the experimental tasks [21]. Moreover, VR serves as a complementary tool in urban planning and environmental psychological research, providing insights into human behavior in complex environments [27, 54]. It blurs the lines between stated preference surveys and revealed preference surveys, providing new insights on how preferences translate into behavior [24].

#### VR limitations in experimental studies

##### Enhancing generalizability: the need for larger, more diversified samples

Several key factors may have influenced the interpretation and generalizability of findings in VR experiments. Limited sample sizes, with a maximum of 150 participants [24], potentially due to resource-demanding procedures of VR studies [4, 24], require cautious interpretation of statistical significance [45]. To enhance result representativeness, using larger sample sizes defined by minimum sample size estimation is essential. Additionally, greater diversity in participants’ socio-demographics and prior VR experience levels will improve the generalizability of results. Participant age, gender, real-life experiences, and active mobility habits may heavily impact environmental perceptions (i.e., safety [24, 45]) and activity mobility behaviors [51] in VR. Participants' occupations should be more diverse as most studies (78%) recruited from homogeneous groups, primarily students or university employees, which heavily narrowed down the variability in VR experiences [4, 45]. Only 33% of studies reported participants' familiarity with VR which is expected to affect participants’ performance and responses [4, 21, 25, 46, 48, 54]. Experienced participants are less likely to be influenced by immersive quality, but more sensitive to graphical imperfections [4, 21], whereas non-experienced participants reported slower movement likely due to novelty [25].

##### Technological limitations: toward enhanced realism and reduced cybersickness

Technology-oriented limitations in VR experiments include cybersickness, limited exposure duration and number of trials [4, 21, 24, 54], low display resolution and field-of-view [4, 50], and challenges in producing high-quality realistic simulations [4, 21]. Cybersickness, experienced in 39% of studies, was associated with negative affective responses [50], and is influenced by factors such as gender, exposure time, content, level of control, and VR type (e.g., 360˚ videos vs. 3D models)[62, 65]. Minimizing cybersickness can be achieved by taking frequent breaks between VR sessions, maintaining high frame rates, ensuring better virtual content quality, creating realistic VEs that match sensory expectations, avoiding high-temperature lab environments, and keeping latency (i.e., delay between user input and the visual response in the VR display) below 20 ms [62]. Interestingly, physical motion in VR may reduce cybersickness compared to navigating through controllers [65], although using a treadmill occasionally led to negative emotions [50].

VR experiment further involves numerous challenges. Replicating weather variations [50] and certain sensory cues (e.g., temperature, wind speed), is scarcely performed although feasible within controlled lab settings, and is expected to affect the sense of presence [54]. Sensations beyond visual and auditory stimuli, such as olfactory (smell) [21, 50] and haptic feedback, especially in a crowded environment [26], play important roles in perception, cognition, and memory [66], but are still a significant technological challenge [4]. Additional challenges include accurately calibrating motion trackers and walking and cycling simulators used to collect data on walking distance, direction, duration, cycling speed, etc. [4]. Additionally, while VR is well-suited for exploring the momentary exposure effect of geographical environments on people's attitudes and behaviors, little is known about the effects of longer and/or repeated VR environmental exposures over time.

### Research agenda

In consideration of future research directions based on identified gaps, four categories are described in Table 10: (a) geographical environment attributes, (b) active mobility behavior, (c) generalizability of findings, and (d) technology-related developments.

**Table 10:** Research agenda for future studies

Category	Research gap	Research agenda
Geographical environment attributes	Lack of complex representation of realistic geographical environment attributes	Incorporating new and more diverse environmental attributes Integrating more complex combinations of attributes
	Lack of diversity in built environments	Incorporating 5Ds including density (e.g., building density), diversity (e.g., mixed land-use), design (e.g., street connectivity, sidewalk connectivity, and path width), distance (e.g., distance to services), and destination (availability of urban facilities)
	Lack of diversity in natural environments	Exploring green environments based on their volume, height (trees, shrubs, grass), emplacement (location on streets), and various types such as green roofs, facades, etc
	Lack of diversity in social environments	Considering the composition of the crowd in conjunction with density Providing precise pedestrian volume data for comparison purposes Exploring the impact of speed (vehicles and people) on the user’s behavior
	Lack of integrating static and dynamic attributes	Combining static and dynamic attributes More variation between dynamic and static attributes
	Limited evidence on the influence of time of day on individuals' perception of their environment	Investigating the effects of time of the day on environmental perceptions
	Lack of knowledge on long-term effects of exposure to geographical environment attributes	Integrating momentary experiences from IVEs with daily routine data (i.e., active mobility habits) to gain insights into potential long-term effects Designing longitudinal VR experiments with follow-up assessments
Active mobility behavior	Limited methods integrating both objective and subjective measurements	Measuring active mobility both objectively and subjectively to achieve a comprehensive understanding of an individual's activity levels, behaviors, perceptions, and affective response
	Lack of exploration into how different destinations and trip purposes affect walking/cycling behavior	Investigating the influence of different destinations, purposes, and travel times on individuals' active behavior [67] (e.g., necessary activities, optional activities, and social activities [68])
Generalizability of the findings	Limited sample sizes	Enhancing research generalizability and reliability by incorporating a larger sample size
	Relying exclusively on convenience sample (i.e., students, colleagues)	Considering diverse composition of participants with different occupational background
	Lack of exploration into diverse socio-economic and socio-cultural groups	Considering diverse socio-economic status groups Considering diverse ethnic groups Conducting comparative analyses with different social groups
Technology-related development	Limited realism and user’s natural interaction with VE	Multidimensionality presentation of VE through incorporating other sensory cues into VR experiences (i.e., sound, smell, or touch) Using higher fidelity scenes and VR rendering techniques to address cyber sickness
	Limited knowledge on the impact of the level of familiarity with VR on perception and behaviors	Reporting and controlling for level of familiarity with VR
	Limited knowledge on the factors contributing to cybersickness and its’ intensity	Reporting on occurrence and intensity of cybersickness Reporting on strategies used to minimize the impacts
	Limited information on the level of immersion and presence and their impact on perception and behaviors	Evaluating the level of immersion and presence achieved in VEs using presence questionnaires, eye tracking and physiological measurements

## Conclusions

Greenness was the most investigated static environmental attribute and heavily contributed to emotional states. Crowd density, as the most common dynamic attribute, significantly influenced people's movement behavior. Future research in VR experiments will need to investigate more diverse attributes of environmental dimensions, as well as how more complex combinations of these attributes, including both static and dynamic ones, may influence people's active behaviors and attitudes. Furthermore, larger and more diverse sample should be included to ensure the generalizability of the findings.

VR experiments allow researchers to manipulate diverse compositions of various geographical environmental attributes under controlled experimental conditions, ensuring both high experimental and ecological validity. Integrating more systematically objective (i.e., wearable devices) and subjective (i.e., questionnaires) measurements of active mobility will provide comprehensive understanding of individuals' active mobility behaviors.

### Supplementary Information

Supplementary Material 1.

## Data Availability

Not applicable.

## Abbreviations

VR: Virtual Reality
VE: Virtual Environment
HMD: Head Mounted Display
CAVE: Computer Aided Virtual Environment
ECG: Electrocardiogram
AHS: Adaptive headlight systems
HR: Heart rate
