# Supplementary material for: The use of Virtual Reality (VR) to assess the impact of geographical environments on walking and cycling: a systematic literature review
Source: Int J Health Geogr. 2024 Jun 8;23:15. doi: 10.1186/s12942-024-00375-6 (PMC11162039; doi:10.1186/s12942-024-00375-6)
Supplement: Supplementary file 1 — Supplementary Material 1. [file 12942_2024_375_MOESM1_ESM.pdf]

Appendix 1:

Appendix1-Table 1. Systematic search terms

**Virtual Reality, its synonyms and types**	"Virtual Reality" OR "Immersive environment" OR "Immersive visualization environment" OR "Virtual environment" OR "Walking simulator" OR "Mixed Reality" OR "VR simulation" OR "Immersive virtual environment" OR "Immersive virtual reality" OR "Head-mounted display" OR "HMD" OR "CAVE" OR "Gamification" OR "360° video" OR "360° environment" OR "Virtual reality exposure" OR "VR system"
**Active mobility behaviour, its synonyms and types**	"Active behaviour" OR "Active behavior" OR "Walking" OR "Cycling" OR "Bicycling" OR "Physical activity" OR "Pedestrian Behaviour" OR "Pedestrian behavior" OR "walking behaviour" OR "walking behaviors" OR "exercise behavior" OR "exercise behaviour" OR "leisure-time physical activity" OR "pedestrian"
**Geographical environments, its synonyms and types**	"Built environment" OR "Outdoor environment" OR "Pedestrian pathway" OR "Public places" OR "Urban space" OR "Sidewalk" OR "Neighbourhood" OR "Neighborhood" OR "Footpath" OR "City" OR "Cities" OR "Walkable environment" OR "Open space" OR "Physical environment" OR "Greenness" OR "Nature" OR "Urban environment" OR "Street environment" OR "Cycle route" OR "Cycle path" OR "Cycle trail" OR "cycle lane" OR "Biklane" OR "Bik path" OR "cycleway" OR "cycling infrastructure" OR "Walk trail" OR "Walk path" OR "Pedestrian trail" OR "Walkability" OR "Bikeability"
**Exclusion criteria**	"Automated vehicle" OR "Autonomous vehicle" OR "Autonomous driving" OR "Driving simulator" OR "Driving performance" OR "Assisted driving" OR "Indoor" OR "Indoor environment" OR "Shopping mall" OR "Military" OR "War" OR "Disease" OR "Rehabilitation" OR "Patient" OR "Injury" OR "Injuries" OR "Nutrient" OR "Education" OR "Training" OR "Gym" OR "Exit" OR "Therapy" OR "height" OR "fitness" OR "Evacuation" OR "Hospital"

Appendix1-Table 2. Data extraction headings

Heading	Data Extracted
**General information**	ID (DOI)<br>Title of article<br>Authors<br>Publication year<br>Sources of data (e.g. journal article, conference paper)<br>The Country where the study was conducted
**Population Characteristics**	Population demographics and baseline characteristics:<br>Gender<br>Age range (adults +18)<br>Experience with VR?<br>Socio-economic status<br>Ethnicity
**Study design**	Randomised or non-randomized<br>Control group: within subject-comparison or between-subject<br>Other study designs
**Intervention group(s) and control group(s)**	Number of withdrawals, exclusions, lost to follow-up and reasons
**Session's characteristics**	Number of sessions per experiment<br>Session characteristics / Time of measurements (baseline, after, during)<br>Length of exposure<br>Duration of experiment /duration of the session
**Active mobility measurements**	Type of active mobility: walking or cycling<br>Subcategory of walking or cycling (health, crossing, navigation, wayfinding)<br>Purpose of walking (destination)<br>Attributes of active mobility measuring<br>Additional variables related to active mobility
**Environment measurements**	Nature or built environment<br>Environmental attribute
**VR measurements**	Type of VR: CAVE or HMD<br>Model<br>Other technologies combined with VR<br>Self-report or objective<br>The level of interaction (natural interaction) of people with VR<br>The degree of immersion/reality (sound, frame rate, resolution, degree of freedom, etc.)<br>Length of exposure<br>Properties of measure (reliability and validity where available)
**Statistical analysis**	Descriptive or inferential<br>Univariate or multivariate<br>Confounding factor
**Results**	Impacts of geographical environments associated with active mobility on people<br>VR pros and cons<br>VR sickness<br>Other results<br>Possible biases
**Conclusion**	Recommendation for future studies<br>Limitations

Appendix1-Table 3. Checklist for assessing the quality of quantitative studies

Criteria		YES (2)	PARTIAL (1)	NO (0)	N/A
**1**	Question / objective sufficiently described?				
**2**	Study design evident and appropriate?				
**3**	Method of subject/comparison group selection or source of information/input variables described and appropriate?				
**4**	Subject (and comparison group, if applicable) characteristics sufficiently described?				
**5**	If interventional and random allocation was possible, was it described?				
**6**	If interventional and blinding of investigators was possible, was it reported?				
**7**	If interventional and blinding of subjects was possible, was it reported?				
**8**	Outcome and (if applicable) exposure measure(s) well defined and robust to measurement / misclassification bias? Means of assessment reported?				
**9**	Sample size appropriate?				
**10**	Analytic methods described/justified and appropriate?				
**11**	Some estimate of variance is reported for the main results?				
**12**	Controlled for confounding?				
**13**	Results reported in sufficient detail?				
**14**	Conclusions supported by the results?				

Appendix1-Table 4. Models of HMD or VR glasses and CAVE setups

HMD	CAVE
HTC Vive (1–3)	MVision 400 Cine 3D projectors (4)
HTC Vive Pro (5–8)	
HTC Vive Pro Eye (9)	
Oculus Rift (10–13)	
Samsung Gear VR (14)	
Samsung Odyssey (12)	
nVisor SX (15)	
VRLogic V82 (16)	
Fove HMD (17)	

References

1. Nazemi M, Van Eggermond MAB, Erath A, Schaffner D, Joos M, Axhausen KW. Studying bicyclists' perceived level of safety using a bicycle simulator combined with immersive virtual reality. Accident Analysis & Prevention. 2021 Mar;151:105943.

2. Lhuillier S, Piolino P, Nicolas S, Gyselinck V. "Run to the hills": Specific contributions of anticipated energy expenditure during active spatial learning. Quarterly Journal of Experimental Psychology. 2022 Dec;75(12):2287–307.

3. Kern AC, Ellermeier W, Jost L. The influence of mood induction by music or a soundscape on presence and emotions in a virtual reality park scenario. In: Proceedings of the 15th International Audio Mostly Conference [Internet]. Graz Austria: ACM; 2020 [cited 2023 Nov 28]. p. 233–6. Available from: https://dl.acm.org/doi/10.1145/3411109.3411129

4. Subramanian LD, O'Neal EE, Roman A, Sherony R, Plumert JM, Kearney JK. How do pedestrians respond to adaptive headlamp systems in vehicles? A road-crossing study in an immersive virtual environment. Accident Analysis & Prevention. 2021 Sep;160:106298.

5. Koilias A, Nelson M, Gubbi S, Mousas C, Anagnostopoulos CN. Evaluating Human Movement Coordination During Immersive Walking in a Virtual Crowd. Behavioral Sciences. 2020a;10(9):130.

6. Koilias A, Nelson MG, Anagnostopoulos C, Mousas C. Immersive walking in a virtual crowd: The effects of the density, speed, and direction of a virtual crowd on human movement behavior. Computer Animation & Virtual. 2020b;31(6):e1928.

7. Koilias A, Mousas C, Anagnostopoulos C. I feel a moving crowd surrounds me: Exploring tactile feedback during immersive walking in a virtual crowd. Computer Animation & Virtual. 2020c;31(4–5):e1963.

8. Chan SHM, Qiu L, Esposito G, Mai KP, Tam KP, Cui J. Nature in virtual reality improves mood and reduces stress: evidence from young adults and senior citizens. Virtual Reality [Internet]. 2021 Nov 26 [cited 2023 Nov 28]; Available from: https://link.springer.com/10.1007/s10055-021-00604-4

9. Birenboim A, Ben-Nun Bloom P, Levit H, Omer I. The Study of Walking, Walkability and Wellbeing in Immersive Virtual Environments. IJERPH. 2021 Jan 6;18(2):364.

10. Birenboim A, Dijst M, Ettema D, De Kruijf J, De Leeuw G, Dogterom N. The utilization of immersive virtual environments for the investigation of environmental preferences. Landscape and Urban Planning. 2019 Sep;189:129–38.

11. Baran PK, Tabrizian P, Zhai Y, Smith JW, Floyd MF. An exploratory study of perceived safety in a neighborhood park using immersive virtual environments. Urban Forestry & Urban Greening. 2018 Oct;35:72–81.

12. Wirth TD, Warren WH. Robust Weighted Averaging Accounts for Recruitment Into Collective Motion in Human Crowds. Front Appl Math Stat. 2021 Nov 3;7:761445.

13. Simeone AL, Mavridou I, Powell W. Altering User Movement Behaviour in Virtual Environments. IEEE Trans Visual Comput Graphics. 2017 Apr;23(4):1312–21.

14. Calogiuri G, Litleskare S, Fagerheim KA, Rydgren TL, Brambilla E, Thurston M. Experiencing Nature through Immersive Virtual Environments: Environmental Perceptions, Physical Engagement, and Affective Responses during a Simulated Nature Walk. Front Psychol. 2018 Jan 23;8:2321.

15. Iachini T, Coello Y, Frassinetti F, Ruggiero G. Body Space in Social Interactions: A Comparison of Reaching and Comfort Distance in Immersive Virtual Reality. Kotz S, editor. PLoS ONE. 2014 Nov 18;9(11):e111511.

16. Nordahl R. Evaluating Environmental Sounds from a Presence Perspective for Virtual Reality Applications. EURASIP Journal on Audio, Speech, and Music Processing. 2010;2010:1–10.

17. Berton F, Hoyet L, Olivier AH, Bruneau J, Le Meur O, Pettre J. Eye-Gaze Activity in Crowds: Impact of Virtual Reality and Density. In: 2020 IEEE Conference on Virtual Reality and 3D User Interfaces (VR) [Internet]. Atlanta, GA, USA: IEEE; 2020 [cited 2023 Nov 28]. p. 322–31. Available from: https://ieeexplore.ieee.org/document/9089637/
